# Cross-sectional description of nursing and midwifery pre-service education accreditation in east, central, and southern Africa in 2013

**DOI:** 10.1186/s12960-017-0224-1

**Published:** 2017-07-24

**Authors:** Carey F. McCarthy, Jessica M. Gross, Andre R. Verani, Annette M. Nkowane, Erica L. Wheeler, Thokozire J. Lipato, Maureen A. Kelley

**Affiliations:** 1 Health Systems and Human Resources Team, Division of Global HIV/TB, U.S. Centers for Disease Control and Prevention, Atlanta, GA United States of America; 2 Health Workforce Department, World Health Organization, Geneva, Switzerland; 3 Pan American Health Organization, World Health Organization, St. Michael, Barbados; 4 Monitoring, Evaluation, Investigations and Research, Nurses and Midwives Council of Malawi, Lilongwe, Malawi; 5 Emory University’s Nell Hodgson Woodruff School of Nursing, Atlanta, GA United States of America

## Abstract

**Background:**

In 2013, the World Health Organization issued guidelines, *Transforming and Scaling Up Health Professional Education and Training*, to improve the quality and relevance of health professional pre-service education. Central to these guidelines was establishing and strengthening education accreditation systems. To establish what current accreditation systems were for nursing and midwifery education and highlight areas for strengthening these systems, a study was undertaken to document the pre-service accreditation policies, approaches, and practices in 16 African countries relative to the 2013 WHO guidelines.

**Methods:**

This study utilized a cross-sectional group survey with a standardized questionnaire administered to a convenience sample of approximately 70 nursing and midwifery leaders from 16 countries in east, central, and southern Africa. Each national delegation completed one survey together, representing the responses for their country.

**Results:**

Almost all countries in this study (15; 94%) mandated pre-service nursing education accreditation However, there was wide variation in who was responsible for accrediting programs. The percent of active programs accredited decreased by program level from 80% for doctorate programs to 62% for masters nursing to 50% for degree nursing to 35% for diploma nursing programs. The majority of countries indicated that accreditation processes were transparent (i.e., included stakeholder engagement (81%), self-assessment (100%), evaluation feedback (94%), and public disclosure (63%)) and that the processes were evaluated on a routine basis (69%). Over half of the countries (nine; 56%) reported limited financial resources as a barrier to increasing accreditation activities, and seven countries (44%) noted limited materials and technical expertise.

**Conclusion:**

In line with the 2013 WHO guidelines, there was a strong legal mandate for nursing education accreditation as compared to the global average of 50%. Accreditation levels were low in the programs that produce the majority of the nurses in this region and were higher in public programs than non-public programs. WHO guidelines for transparency and routine review were met more so than standards-based and independent accreditation processes. The new global strategy, Workforce 2030, has renewed the focus on accreditation and provides an opportunity to strengthen pre-service accreditation and ensure the production of a qualified and relevant nursing workforce.

**Electronic supplementary material:**

The online version of this article (doi:10.1186/s12960-017-0224-1) contains supplementary material, which is available to authorized users.

## Background

The 2006 World Health Assembly called for an unprecedented scale-up of health worker training [1]. Among the global responders was the US President’s Emergency Plan for AIDS Relief (PEPFAR) with a commitment to support the training and retention of 140 000 new healthcare workers, primarily in sub-Saharan Africa [2–5]. Subsequent global policy documents from the World Health Organization (WHO) echoed the need to increase the number of health workers but also to ensure new health workers are part of a skilled and competent workforce that can promote universal access to health services, including antiretroviral therapy (ART) to treat and prevent human immunodeficiency virus (HIV) transmission [2, 6, 7]. The landmark Lancet Commission Report (2010), *Health professionals for a new century: transforming education to strengthen health systems in an interdependent world*, identified accreditation of health professional education institutions as an essential prerequisite for quality health professional education [8]. Lastly, the WHO Global Strategy on Human Resources for Health: Workforce 2030 identified accreditation mechanisms for health training institutions as a milestone to be achieved by all countries by the year 2020 [9].

The World Federation for Medical Education (WFME) defines accreditation as “a process of review and approval by which an institution or program is granted a time-limited recognition of having met certain established standards” [10]. Accreditation can be an effective tool aligning health professional education with population health needs to ensure new graduates have the relevant competencies and skills to address current public health requirements [11]. In doing so, accreditation systems can also help strengthen social accountability in national health professional education [12, 13]. Innovative approaches in health professional education, and a growing demand for accountability and quality assurance in higher education, have contributed to a stronger focus on accreditation worldwide [8]. However, less than half of the countries globally have a comprehensive accreditation system that is credible and transparent; most have systems where programs and schools are either not reviewed or reviews are conducted on an ad hoc basis [14].

In 2013, WHO, released guidelines for transforming and scaling up health professional education and training [11]. The guidelines set out a vision for increasing the quantity, quality, and relevance of health professionals and provide recommendations on how to produce health professional graduates equipped to meet the needs of the communities they serve [11]. “Accreditation and regulation” is one of the five domains of the guidelines. A specific recommendation is for governments to introduce or strengthen health professional education accreditation [11]. Key considerations for governments include ensuring the accreditation process is transparent, based on standards, supported by legislation and undertaken independently, and that the process is periodically evaluated [14]. According to WHO policy briefs developed as part of the guideline process, carrying out successful accreditation requires the requisite human, material, and financial resources, as well as periodic evaluation of the system itself [14]. The systems need to be legally legitimate with the authority to accredit and sanction, ensuring that the accreditation process is efficient and includes at the very least a self-assessment, external review, site visit, and assessment report [14]. To effectively monitor national accreditation practices, WHO recommends data collection by the professional regulatory bodies, such as accrediting agencies or health professional councils, employers, and health professional training institutions [11].

While country-level reviews of pre-service nursing education in the African region have been published [15], there has not yet been a systematic assessment of nursing and midwifery pre-service accreditation in the East, Central, and Southern Africa (ECSA) region. This study sought to gain an understanding of current pre-service accreditation policies, approaches, and practices in 16 ECSA countries relative to the 2013 WHO guidelines, *Transforming and Scaling Up Health Professional Education and Training.*

## Methods

This study utilized a cross-sectional group survey with a standardized questionnaire administered to a convenience sample of approximately 70 nursing and midwifery leaders from 16 countries in attendance at the PEPFAR-supported African Health Profession Regulatory Collaborative (ARC) [16–18] forum from July 30 to August 2, 2013, in Nairobi, Kenya. The countries represented at the ARC meeting included Botswana, Ethiopia, Kenya, Lesotho, Malawi, Mauritius, Mozambique, Namibia, Rwanda, Seychelles, South Africa, South Sudan, Swaziland, Tanzania, Uganda, Zambia, and Zimbabwe. For each country, the leaders included the chief nursing officer in the ministry of health, registrar of the nursing and midwifery council, head of the professional nursing and midwifery association, and a representative from a nursing or midwifery academic institution. Incentives were not provided to participate in the study.

Data were collected using a standardized survey (Additional file 1) developed by the authors in 2013. Survey questions included the number and level of pre-service nursing and midwifery training schools or programs and the systems, approaches, and mechanisms of accreditation of pre-service nursing and midwifery education, as well as the challenges associated with accrediting nursing and midwifery training programs. Each country delegation of nursing leaders completed one survey together, representing the responses for their country (i.e., a group survey). A group survey was deemed superior to an individual survey to avoid the possibility of missing or conflicting information within the same country. Surveys were administered and completed in English for all countries, including the Mozambican group which was assisted by a professional Portuguese-English translator. The survey included closed-choice questions with comment boxes for the choice of “other,” Likert scale questions, and open-ended questions with special instructions for what participants should do if there is no consensus among group members on a particular response. The U.S. Centers for Disease Control and Prevention reviewed and approved the study protocol.

The surveys were identified by country; professional positions represented by survey respondents were noted. All the data were entered and stored in a password-protected database to facilitate confidentiality and consistent analysis by select co-authors across countries. Missing data or data requiring verification were collected during the conference or via email to ensure the completeness and trustworthiness of the data for analysis. Each individual country served as the unit of analysis, providing a composite description of the nursing and midwifery pre-service training programs, the accreditation systems and approach, and country-level accreditation challenges faced by nursing regulatory bodies. The definition of accreditation used on the survey was the following: “A process of review and approval by which an institution is granted time-limited recognition of having met certain established standards” [9]. Midwifery programs are defined as midwifery-only programs. Ethiopia, Mozambique, and South Africa’s nursing and midwifery programs and Tanzania’s certificate, diploma, post-basic, and degree programs were excluded from the accreditation status analysis due to missing data.

## Results

Survey responses represented 16 countries, as South Sudan did not participate in the study, with highly variable numbers of nurses in the country, from over 500 nurses per 100 000 population (South Africa) to about 25 per 100 000 (Ethiopia), and a range of program options by country, from basic certificate to an advanced PhD (Table 1). Countries also varied in the number of nursing and midwifery training institutions, from six in Swaziland and the Seychelles to over 100 in Kenya. The greater the number of schools in a country, the more likely they were to have non-public (i.e., private or faith-based) nursing schools as an option for pre-service education, with the exception of Swaziland and Malawi that had relatively few schools, of which 50% were non-public (*n* = 3 and *n* = 8, respectively). Countries in which the majority of schools were public (i.e., government), such as Botswana, Namibia, Rwanda, and Zambia, reported the highest levels of accreditation. Uganda and Zimbabwe reported 100% accreditation of schools across public and non-public institutions. Kenya, Mozambique, and Seychelles reported accreditation levels too low to cover even the public schools.

**Table 1:** Country-level nursing workforce and training descriptive statistics, 2013

Column	1	2	3	4	5
Country	# Nurses in country (nurse to population ratio per 100 000)[19]	# (*N*) of nursing and midwifery programs	% (*N*) of nursing and midwifery programs that are public	% (*N*) of all programs accredited (derived from reported # of schools and # accredited in last 5 years)	Types of programs C = certificate D = diploma PB = post-basic G = degree M = masters PhD = doctorate
Botswana	335	18	100% (18)	100% (18)	D PB G M
Ethiopia	25	Missing data	Missing data	Missing data	D PB G M
Kenya	86	104	51% (53)	19% (20)	C D PB G M
Lesotho^a^	62	10	50% (5)	40% (4)	C D PB G
Malawi	34	16	50% (8)	100% (16)	C D PB G M
Mauritius	373	Missing data	Missing data	Missing data	C D PB
Mozambique	41	8	100% (8)	75% (6)	C PB G M
Namibia	278	11	100% (11)	100% (11)	C D PB G M PhD
Rwanda	69	16	75% (12)	81% (13)	D G
Seychelles	481	6	100% (6)	33% (2)	D PB
South Africa	511	Missing data	Missing data	Missing data	C D PB G M PhD
Swaziland	160	6	50% (3)	0% (0)	C D PB G
Tanzania	44	86	43% (37)	*Missing data*	C D PB G M PhD
Uganda	131	30	53% (16)	100% (30)	C D PB G M
Zambia	78	14	79% (11)	86% (12)	C D PB G M
Zimbabwe	134	25	88% (22)	100% (25)	C D PB G M PhD

^a^Includes nursing assistant category as certificate nurses

Country teams were asked whether accreditation was mandatory, where it was mandated in policy, and which bodies conducted the accreditation. Investigators then consulted the primary sources listed to find national health laws and policies addressing accreditation (Table 2). Accreditation of pre-service nursing and midwifery training institutions was mandated in 15 of the countries surveyed (94%). The nursing council served as the sole accrediting body in five countries, including Kenya, Malawi, South Africa, Zambia, and Zimbabwe. Accreditation was a shared responsibility with another entity in four countries, such as an education council in Lesotho, an independent qualification authority in Namibia and the Seychelles, and the Ministry of Health and Ministry of Education in Uganda. The Ministry of Education accredited nursing institutions in Ethiopia and Rwanda, whereas Mauritius and Swaziland used independent authorities. Botswana and Tanzania relied on educational councils. The mandate for the accreditation of nurse training institutions was located in the national nurses and midwives act for 12 of the 16 countries surveyed (75%).

**Table 2:** Accreditation mandate, body, and health professional legislation by country, 2013

What was reported about accreditation				
Country	Accreditation mandated?	Accrediting body	Where mandated	Language within the pertinent health professional legislation
Botswana	Yes	Tertiary Education Council University of Botswana	- Tertiary education policy - Nursing and MidwiferyCouncil Act - Center for academic development	Nurses and Midwives Act, 1995, Section 7 Duty and powers of the Council: “to approve, subject to inspection, schools of nursing and institutions where student nurses and midwives, and enrolled nurses are trained;”
Ethiopia	Yes	Ministry of Education (in collaboration with a nursing school)	- Has its own policies and regulation using checklists	
Kenya	Yes	Nursing Council of Kenya	- Nurses Act - Accreditation regulations	Nurses Act, 1983, Section 9 Function of the Council: “to recommend to the Minister institutions to be approved institutions for training of persons seeking registration or enrolment under the Act:”
Lesotho	Yes	Lesotho Nursing Council Council of Higher Education	- Revised 2012 Nurses’ Act Accreditation	Nurses and Midwives Act, 1998, Section 4 Functions of the Council: “to recommend to the Minister, the schools of nursing or other places or institutions where student nurses, midwives or other categories of nurses are to be trained;”
Malawi	Yes	Nurses and Midwives Council National Council for Higher Education	- Nurses and Midwives Act No 19 (1995)	Nurses and Midwives Act, 1995, Section 12 Powers of the Council: “approve nursing schools in accordance with the prescribed conditions,”
Mauritius	Yes	Mauritius Qualification Authority	- Central School of Nursing Curriculum development with the awarding body, MQA, Nursing Council, staff unions	The Nursing Council Act, 2003, Section 2: “‘recognised nursing institution’ means a school, university, college, faculty or other similar body which is authorized under the laws of a country to provide courses leading to nursing or midwifery and is prescribed by regulations made by the Minister after consultation with the Council or other relevant bodies;”
Mozambique	No	Ministry of Health Ministry of Education		
Namibia	Yes	Nursing Council of Namibia National Qualifications Authority Senate of University of Namibia	- Nursing Act of 2004 - The National Qualification Act - The University of Namibia Act	Nursing Act, 2004, Section 1: “educational institution’ means any nursing education and training institution, approved by the Council in writing,”
Rwanda	Yes	Ministry of Education (Higher Education Council)	- Yes, but the Ministry of Education does not involve the Nursing and Midwifery council	Law establishing the National Council of Nurses and Midwives, 2008, Article 8 Board of Directors and its responsibilities: “to determine the requirements for the approval of the course program of Nurses and Midwives and to ensure its implementation;”
Seychelles	Yes	Nurses and Midwives Council Seychelles Qualifications Authority	- Nurses and Midwives Act, 1985 - Seychelles Qualification Act, 2005	Nurses and Midwives Act, 1985, Section 15: “the Minister may, after consultation with the council make regulations … providing for courses of training,”
South Africa	Yes	South African Nursing Council	- Nursing Act 33 of 2005	Nursing Act, 2005, Section 1: “‘nursing education institution’ means any nursing education institution accredited by the Council in terms of this Act;”
Swaziland	Yes	Outsourced Body	- Nursing and Midwife Act, 1965	Standards of Nursing Education and Practice: “The government, through the Nursing Act, gives the Swaziland Nursing Council the responsibility to set standards of nursing education and practice as well as to approve nursing education and practice programs in the country.”
Tanzania	Yes	Tanzania Commission for University (TCU) National Council for Technical Education (NACTE)	- TCU, NACTE, and Nurses accredit - (Midwifery Council “verifies”)	Nursing and Midwifery Act, 2010, Section 3: “‘approved nursing institution’ means an institution or part of an institution approved by the Council under the provisions of this Act, to provide a course leading to the acquisition of a qualifying award;”
Uganda	Yes	Nursing Council Ministry of Health Ministry of Education and Sport	- Government Policy	Nurses and Midwives Act, 1996, Section 3 Functions of the Council: “to approve courses of study for nurses and midwives”
Zambia	Yes	Nursing and Midwifery council	- Nurses and Midwives Act	The Nurses and Midwives Act, 1997, Section 4 Functions of the Council: “register training colleges for nurses and midwives”
Zimbabwe	Yes	Nursing Council of Zimbabwe	- Ministry of Health and Child Welfare	Health Professions Act, 2000, Section 42 Functions and powers of Nursing Council: “to regulate, control and supervise all matters affecting the training of persons in, and the manner of the exercise of, the professions and callings specified in Part IV of the First Schedule;”

**Fig. 1:**
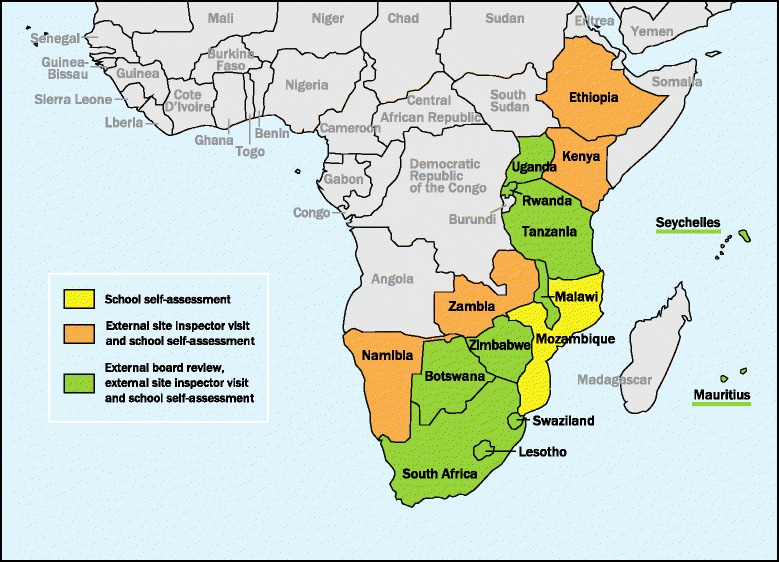
Accreditation process by country, 2013

The reported approaches and processes for accrediting nursing and midwifery institutions varied across countries (Fig. 1). The approach typically involved an institutional self-assessment (16 countries) and the use of external inspectors (15 countries). Eleven countries (69%) used an independent board to review the internal and external assessments and make the accreditation decision. Four countries (25%) utilized self-assessment and external inspection without an independent board review. Fifteen countries (94%) provided feedback to programs and schools regarding their accreditation evaluations. The accreditation processes varied. In 14 countries, different levels of accreditation were granted; 13 countries involved stakeholders in the process; and 10 countries required nursing and midwifery programs to renew their accreditation, yet it was only enforced in seven of the countries. Twelve countries (86%) reported their accreditation process was based on standards, with 10 countries using national standards, three using regional standards, and one using international standards.

The proportion of programs accredited varied across program levels and types (Table 3). The percentage of accreditation for active nursing programs decreased by program level from 80% for doctorate programs to 62% for masters nursing to 50% for degree nursing to 35% for diploma nursing programs. Diploma nursing programs represented 37% of the reported 350 active programs; however, only 35% of diploma nursing programs were accredited. Accreditation was lower among non-public nursing diploma (24%) and degree (33%) programs compared to public nursing diploma (44%) and degree (58%) programs. While midwifery programs only represented 14% of the active programs, a higher percent of midwifery programs were accredited at the certificate (100%) and diploma (91%) levels.

**Table 3:**
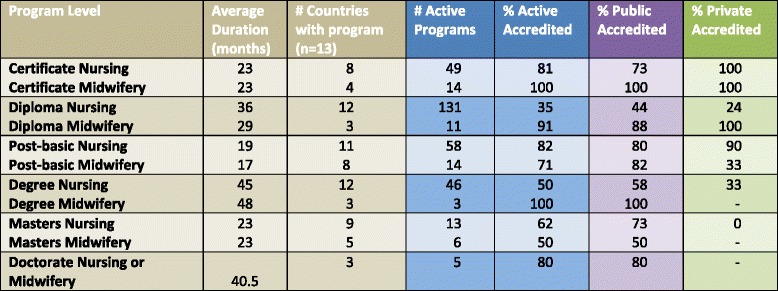
Duration, number, and accreditation status of nursing and midwifery programs by level, 2013

**Fig. 2:**
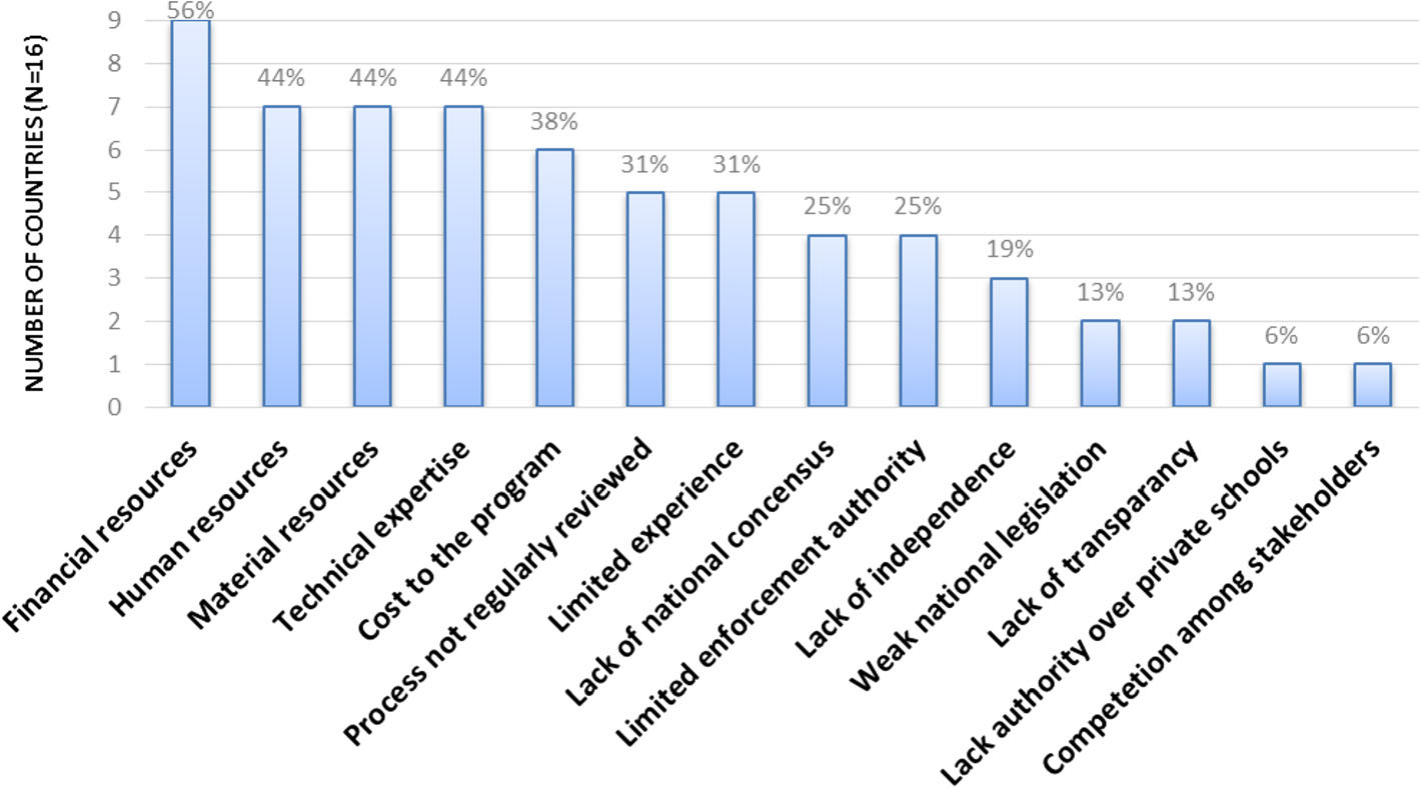
Most frequently cited challenges to expanding accreditation, 2013

Countries were asked to rate whether or not certain issues were challenges to them in carrying out accreditation processes (Fig. 2). Nine of the countries (56%) reported limited financial resources to coordinate or carry out accreditation site visits as a serious challenge. Seven countries (44%) reported limited human, material, and technical expertise related to accreditation inspections as key challenges. Six countries (38%) noted challenges due to the cost to the program being accredited, and five countries (31%) identified limited experience of the accrediting body and no consistent review of the accreditation process. As for key considerations for accreditation from WHO, 14 countries (87%) reported there were no challenges with national legislation or transparency in the accreditation process and 15 countries (94%) reported no challenges with their authority to accredit non-public institutions or competition among stakeholders.

The guidelines also recommended that accreditation be transparent and the process be evaluated periodically [11]. Issues of transparency included stakeholder engagement, self-assessment, evaluation feedback, and public disclosure. Thirteen countries (81%) considered stakeholder input when establishing the national accreditation standards and process, 16 countries (100%) utilized a self-assessment, 15 countries (94%) provided feedback to the institution concerning their accreditation evaluation, and 10 countries (63%) disclosed schools’ and programs’ accreditation status to the public, indicating higher levels of transparency in the establishment of accreditation standards and feedback to institutions under review than in public disclosure of institutions’ accreditation results. Eleven countries (69%) reported that they regularly reviewed their standards and process for accreditation, highlighting the need for more routine reviews of national accreditation systems.

## Discussion

This study provided a baseline assessment of nursing and midwifery pre-service education accreditation in 16 countries in the ECSA region with respect to the 2013 WHO guidelines [11]. The guidelines recommended strengthening health professional accreditation supported by legislation [11]. Fifteen of the 16 ECSA countries surveyed (94%) mandate accreditation of pre-service education, which is a higher percentage than the global estimate of 50%. Two of the essential components of an accreditation process are met to a high degree, including self-assessments required by all 16 countries (100%) and external reviews in 15 countries (94%). However, even though most countries mandate accreditation, only 35% of the 131 diploma nursing programs, which produce the majority of the nursing workforce in the ECSA region [15], are accredited. The percent accreditation is stronger for public nursing diploma (44%) and degree (58%) programs compared to non-public nursing diploma (24%) and degree (33%) programs. Until countries are able to increase the percentage of diploma programs that are accredited, especially at non-public schools, it will be difficult to ensure these students receive a consistent, high quality education.

The WHO guidelines recommend accreditation be based on standards and done independently [11]. Twelve countries (75%) reported that their accreditation process is based on national, regional, or international standards, or a combination thereof. Five countries (31%) do not require the use of external board review and only seven countries (44%) enforce routine renewal of accreditation for pre-service training programs—steps countries could incorporate to enhance accreditation. While seven countries (44%) use a body other than the nursing council as the accrediting body, the role of the nursing council in accreditation should be evaluated to ensure its professional contribution to the accreditation of nursing and midwifery programs, which is often mandated in the national nursing act. In instances where the council is not the direct accrediting body, councils could consider providing technical tools for institutional self-assessments, external inspection score cards, and guidelines for routine accreditation renewal among other guidance to the accrediting bodies. Nine countries (56%) noted financial challenges, and seven (44%) identified challenges with human, material, and technical resources. Administration fees charged to institutions and programs undergoing the accreditation process can be utilized to garner additional resources, which could continue to increase as routine accreditation renewals are expanded, to support strengthening the accreditation process.

Despite the recent focus on and support for health professional training in low- and middle-income countries [15–17], many in sub-Saharan Africa still face substantial challenges to producing a higher quantity, quality, and relevance of health professionals, particularly given high levels of health professional migration, proliferation of private and for-profit educational institutions, and dynamic population health needs [1, 18–20]. Substantial gaps in the number of faculty, quality of curricula, health professional regulation, and available resources will likely make meeting the new accreditation recommendations difficult [9, 15, 21, 22]. The 2015 PEPFAR human resources for health strategy calls for an “adequate supply and quality of human resources for health to expand HIV/AIDS services,” highlighting the need for periodic review of pre-service training curricula as a part of the accreditation review process [20]. The WHO Global Strategy on Human Resources for Health: Workforce 2030 made accreditation a key global milestone; countries must report annually on the presence of established accreditation mechanisms for health training institutions [9]. Particularly pertinent to our study sample, Workforce 2030 also recommends that health professional regulatory councils institute greater oversight of accreditation activities.

The limitations of this study include the biases intrinsic in self-report surveys, including recall bias and social desirability bias. This study also used convenience sampling, which may not represent the entire population of interest. In addition, questions did not address a main recommendation within the WHO guidelines: whether or not the government was currently supporting efforts to develop or strengthen accreditation systems. Lastly, this study was conducted in late 2013, and certain elements of country-level education or accreditation may have changed since the data were collected. Despite the limitations, the findings of this study will be particularly relevant in the African region, where the WHO regional office has embarked on the development of prototype curricula for pre-service nursing and midwifery programs [21], as well as a regional professional regulatory framework [22]. The information from this study should be useful as these tools are piloted in the Africa region. Furthermore, the study establishes a baseline of countries’ pre-service education accreditation systems and provides a benchmark from which to measure progress towards global milestones set by WHO for 2020 and 2030.

## Conclusion

The process of accreditation can certify to what extent schools responsible for educating health professionals meet quality standards to ensure graduates obtain core competencies [14]. This study of nursing and midwifery pre-service accreditation in 16 sub-Saharan African countries presents a description of current policies, practices, and approaches relative to the 2013 WHO guidelines to strengthen health professional education for the new century. Efforts to strengthen accreditation for nursing and midwifery pre-service training institutions should focus on reviewing countries’ national accreditation process and standards, utilizing an external board review in the process, increasing accreditation for diploma programs and private programs, and enforcing routine renewal of programs’ accreditation status. Given the challenging context in which countries are striving to meet the ambitious global milestones set by Workforce 2030, targets to improve accreditation will require increasingly coordinated policy investments in the health workforce nationally and globally.

## Additional file

Additional file 1: Accreditation survey tool. (DOCX 268 kb)

## Abbreviations

ARC: African Health Professions Regulatory Collaborative
ART: Antiretroviral therapy
ECSA: East, Central, and Southern Africa
HIV/AIDS: Human immunodeficiency virus/acquired immune deficiency syndrome
PEPFAR: President’s Emergency Plan for AIDS Relief
WFME: World Federation for Medical Education
WHO: World Health Organization
