# Supplementary material for: Cross-sectional description of nursing and midwifery pre-service education accreditation in east, central, and southern Africa in 2013
Source: Hum Resour Health. 2017 Jul 24;15:48. doi: 10.1186/s12960-017-0224-1 (PMC5525227; doi:10.1186/s12960-017-0224-1)
Supplement: Additional file 1: — Accreditation survey tool. (DOCX 268 kb) [file 12960_2017_224_MOESM1_ESM.docx]

**Appendix 1: Accreditation Survey Tool**
